# Supplementary material for: Lexical-semantic properties of verbs and nouns used in conversation by people with Alzheimer’s disease
Source: PLoS One. 2023 Aug 3;18(8):e0288556. doi: 10.1371/journal.pone.0288556 (PMC10399788; doi:10.1371/journal.pone.0288556)
Supplement: Attachment — Submitted filename: Response to Reviewers.docx [file pone.0288556.s001.docx]

Dear Dr. Hand,

**Manuscript number: PONE-D-23-03295**

**Title: Lexical-semantic properties of verbs and nouns used in conversation by people with Alzheimer's disease**

Please find attached a revised copy of the above-referenced manuscript. We would like to thank both reviewers as well as yourself for your encouraging feedback and the very helpful and concise suggestions for improvement. We feel that this commentary has significantly enhanced the quality of the manuscript.

All editor and reviewer suggestions are listed in Sections A to C below in italics, along with information in blue text on how these have been addressed. Where line numbers have changed, we provide updated ones, always referring to the unmarked version. The attached revised manuscript reflects the implementation of suggestions using tracked changes.

Other tracked changes include the following:

- To support information provided in the data availability statement and address a point made in the decision letter (please see below), we have added within the manuscript a link to the corpus that houses the data analysed.
- Acknowledgments have been updated to reflect an academic promotion.
- The reference list now includes information for two studies that had previously been cited within the text but did not appear in referencing (numbers 58 and 59). Also, as a result of the editor’s suggestion to augment the literature review, two references have been added (numbers 2 and 3). One of these replaces a previously used one —the Reed et al., 2016 one—as the new reference supported the corresponding text while allowing for expansion.

In its postscript, the decision letter specifies the need to address the following additional requirements:

1. Style requirements:

We have revised our initial format-free submission to ensure that it complies with PLOS ONE formatting requirements for publication. We have not tracked these revisions, as they include no changes to content. Formatting revisions include the following:

- changes to title page
- changes to heading levels and font sizes of headings and removal of heading numbers
- change from APA to Vancouver referencing for in-text citations and reference list
- use of lettered panel labels in the caption of a figure with panels (Fig 2)
- revision of figures using the PACE tool to ensure that they meet PLOS ONE requirements

2. Minimal underlying dataset:

The decision letter describes the need, when submitting to PLOS ONE, to provide the minimal underlying dataset—except in cases where there are legal or ethical restrictions on sharing data publicly. Such restrictions apply to the third-party data we have used. Further information on those restrictions can be found at the corpus website, which is now provided within the manuscript text. We describe this rationale in our submitted data availability statement, which we include here for ease of reference:

We are unable to make our data publicly accessible, as the data originate from a third-party source and are not owned by us. Our data originate from the Carolina Conversations Collection (CCC), <https://carolinaconversations.musc.edu/ccc/about/>. This is a digital archive of recorded interviews with American men and women aged 65 and older, many of whom are experiencing cognitive decline or other health issues. Our study involved analysis of speech samples from the CCC, and our data files include all words produced in the speech samples analysed. Even with that information removed from our data files, speech samples can be rebuilt wholly or in part using data relevant to the reported study. Researchers can gain access to the CCC by following the process described here: <https://carolinaconversations.musc.edu/ccc/help/access/approval>. Once researchers have been granted CCC access, reasonable requests for our study-specific data can be directed to any of the study’s authors.

Please be in touch if further information is needed.

3. Reference list:

This point specifies the need to review the reference list to ensure that it is complete and correct and to mention any changes in our response letter. Changes to the reference list are discussed above and have resulted in a net gain of 3 studies. Those changes are tracked. The list is also now in Vancouver style, a change that is not tracked.

A. **Editor’s comments:**

*I hope that you find the reviewer's comments helpful. Additionally, I would recommend that you consider augmenting certain sections of your literature review to include the work of, for example, Boyd Davis, Paul Darden, et al.*

We found the reviewers’ comments extremely helpful.

We also appreciated your suggestions of references to help establish the scientific context for our work. We identified two studies by Boyd Davis and colleagues that presented ramifications of changes to language and communication in Alzheimer’s disease. These now appear in the text and reference list as references 2 and 3. Use of these resources has resulted in changes to lines 39-40 (unmarked version) of the Introduction. Reference number 2 also supports and allows for expansion on a point made in the Discussion section; as a result, changes were also made to lines 583-584.

B. **Reviewer 1’s comments**

*the new standard term is /people living with dementia/ -- I don't think, from personal observation when collecting a lot of the data in the collection, that they ALL had Alzheimer's. Your abbreviation throughout should be PLWD*

We acknowledge Reviewer 1’s familiarity with the CCC. We are happy to make this change if necessary, pending confirmation of whether the reviewer’s comment considers our description of inclusion criteria in the “Participants and language samples” section. The CCC includes Alzheimer’s disease and dementia as two distinct selection criteria under the Condition heading. We specifically included only data from participants meeting the Alzheimer’s criterion—no one who was only described using the broader term *dementia*. Our description thus reads, “Interviewees were considered for inclusion in our AD group only if they met the following criteria: … **dementia specified as AD**…” (bold letters added for emphasis). Can the reviewer please clarify whether this comment applies to participants designated specifically in the CCC search filters as having AD? If it does, we are happy to apply this change. If the comment no longer applies, but the reviewer feels our description could be clarified, we are happy to take suggestions on how to do so.

*Set off the hypotheses as a bulleted list or it gets confusing*

We have made this change and agree that it improves readability. Please see lines 95 to 104 of the unmarked version.

C. **Reviewer 2’s Comments**

*Line 74-75 'Interestingly, AoA comparisons have not indicated reliance on simpler words by pwAD' - could the authors please explain what is meant here.*

We have revised and added detail to this statement. It now reads, “Interestingly, findings from picture description tasks have not indicated that pwAD rely on words of lower AoA [8, 19]. Fraser et al. [8] included AoA in their investigation of language features that might help distinguish pwAD from healthy older people but did not find it to contribute to identification of pwAD. Yeung et al. [19], meanwhile, actually found pwAD to use words of higher AoA than controls, a finding they attributed to a tendency by pwAD to produce utterances that were inappropriate to the task.” In the current unmarked version, that information can be found in lines 68 to 73.

*Line 140-141 'Groups were not matched on education because this was provided in 4-year ranges' - could the authors please explain what is meant here.*

We thank the reviewer for highlighting this. In following up on the comment, we found our initial description not to be fully accurate and we have rephrased as: “Groups were not matched for education because the CCC provides this only in broad ranges that do not facilitate meaningful matching—e.g., 1-8 years, 9-12 years, etc.” As a result of the revision process, this information now appears in lines 141-143. We have also revised wording now in line 411, as that sentence reiterates this point on education.

We again thank the editor and reviewers for your time and consideration in regard to this manuscript. We hope that these revisions meet your satisfaction and PLOS ONE’s publication criteria.

Sincerely,

Eric Williams, Catherine Theys, and Megan McAuliffe
